# Adherence to colorectal cancer screening guidelines in Canada

**DOI:** 10.1186/1471-230X-7-39

**Published:** 2007-10-02

**Authors:** Maida J Sewitch, Caroline Fournier, Antonio Ciampi, Alina Dyachenko

**Affiliations:** 1 Department of Medicine, McGill University, Montreal, Canada; 2 Division of Clinical Epidemiology, Research Institute of the McGill University Health Center, Montreal, Canada; 3 Department of Epidemiology and Biostatistics, McGill University, Montreal, Canada; 4 Department of Epidemiology and Community Studies, St. Mary's Hospital Center, Montreal, Canada

## Abstract

**Background:**

To identify correlates of adherence to colorectal cancer (CRC) screening guidelines in average-risk Canadians.

**Methods:**

2003 Canadian Community Health Survey Cycle 2.1 respondents who were at least 50 years old, without past or present CRC and living in Ontario, Newfoundland, Saskatchewan, and British Columbia were included. Outcomes, defined according to current CRC screening guidelines, included adherence to: i) fecal occult blood test (FOBT) (in prior 2 years), ii) endoscopy (colonoscopy/sigmoidoscopy) (prior 10 years), and iii) adherence to CRC screening guidelines, defined as either (i) or (ii). Generalized estimating equations regression was employed to identify correlates of the study outcomes.

**Results:**

Of the 17,498 respondents, 70% were non-adherent CRC screening to guidelines. Specifically, 85% and 79% were non-adherent to FOBT and endoscopy, respectively. Correlates for all outcomes were: having a regular physician (OR = (i) 2.68; (ii) 1.91; (iii) 2.39), getting a flu shot (OR = (i) 1.59; (ii) 1.51; (iii) 1.55), and having a chronic condition (OR = (i) 1.32; (ii) 1.48; (iii) 1.43). Greater physical activity, higher consumption of fruits and vegetables and smoking cessation were each associated with at least 1 outcome. Self-perceived stress was modestly associated with increased odds of adherence to endoscopy and to CRC screening guidelines (OR = (ii) 1.07; (iii) 1.06, respectively).

**Conclusion:**

Healthy lifestyle behaviors and factors that motivate people to seek health care were associated with adherence, implying that invitations for CRC screening should come from sources that are independent of physicians, such as the government, in order to reduce disparities in CRC screening.

## Background

In Canada, colorectal cancer (CRC) is the third most commonly diagnosed cancer in men and women and the second leading cause of cancer deaths [1]. According to the 2006 Canadian Cancer statistics, an estimated 1 in 14 men and 1 in 16 women will develop CRC in their lifetimes and 1 in 28 men and 1 in 31 women will die from CRC. CRC screening reduces both CRC incidence through removal of premalignant polyps and CRC deaths through early detection and treatment. Since 1996, Canadian and U.S. organizations have published guidelines for CRC screening in individuals who are 50 years of age and older and at average-risk for developing the disease [2-6]. Recommendations include performance of either annual or biannual FOBT, flexible sigmoidoscopy every 5 years, double contrast barium enema every 5 to 10 years or colonoscopy every 10 years.

Although routine CRC screening can reduce incidence, morbidity and mortality from the disease, CRC screening in Canada is suboptimal [7-9]. Most (58% to 92%) physicians report recommending CRC screening to average-risk patients [10-12] and undergoing screening themselves, mainly with colonoscopy [13]. But the best case scenario, estimated from survey data, is that 23% of the screen-eligible population has ever been screened [8] and that 53% of Canadian physicians have undergone CRC screening [13]. These physician rates are comparable to rates for the CRC screen-eligible population in the U.S., which range between 38% and 54% [14,15]. Yet there are methodological challenges to estimating population CRC screening rates. The universal access, publicly funded health care systems in the Canadian provinces collect information on performance of large bowel procedures, but do not collect information on use of FOBT. This is problematic because FOBT and colonoscopy are the two procedures most often performed for CRC screening. Surveys, by comparison, commonly supply data on FOBT but rely on self-report. Whereas self-report could be problematic for distinguishing between sigmoidoscopy and colonoscopy, independent studies show good sensitivity and specificity for self-reported procedural use, especially when sigmoidoscopy and colonoscopy are grouped together[16,17].

Health Canada considers CRC ideal for mass screening of average individuals [18]. However, because population-based CRC screening programs are only now, in 2007, being established, most referrals for CRC screening occurred in primary care practices, which are major sites for providing health promotion and screening services. Not surprising, studies consistently report associations between receipt of a physician referral for CRC screening [19-24] and adherence to CRC screening guidelines. Other patient characteristics associated with this outcome include older age[20,25,26], sex[20,22], higher social class[26,27], White race [21,28], having health insurance [26] or usual source of health care[26,29], family history of CRC[30], higher education[26], higher socioeconomic status[26,27,31], knowledge of CRC[19-24,32], perceived risk of developing CRC [19-24] and adhering to other preventive health behaviours[25,30,33].

Given the limited knowledge about CRC screening in Canada and the variability in physician referral behaviour, we sought to evaluate adherence to CRC screening guidelines by utilizing data from a national survey. The purpose of this study was to identify individual and health system level characteristics associated with adherence to CRC screening guidelines in average-risk residents of Newfoundland, Ontario, Saskatchewan and British Columbia. We employed a biopsychosocial framework, which integrates biomedical, psychosocial and environmental factors, to better understand disparities in adherence to CRC screening guidelines. Elucidating the factors associated with adherence to CRC screening guidelines may help tailor interventions aimed at improving utilization of CRC screening.

## Methods

### Data sources

The principal data source for this evaluative population-based study was the Statistics Canada 2003 Canadian Community Health Survey (CCHS) Cycle 2.1. The CCHS targeted persons aged 12 years and older who were living in private dwellings in all Canadian provinces and territories. Exclusions included individuals living on Indian reserves, institutional residents, full-time members of the Canadian Armed Forces and residents of certain remote regions. Survey methods employed either personal or telephone interviews using a questionnaire designed for computer-assisted interviewing and have been describe elsewhere in greater detail[18]. In both interview processes, complex multi-step strategies were used to maximize response rates; an overall national response rate of 80.7% was achieved.

The CCHS Cycle 2.1 survey data consisted of non-nominative information on health status, health determinants and health care utilization. Some information was asked of all respondents, while other information was asked of a sub-sample of respondents large enough to generate reliable provincial and national estimates. Optional information was asked of all respondents in selected health regions. The CRC screening module was optional, and administered to approximately 40,000 individuals in all health regions of Newfoundland and British Columbia and in 14 of 37 and 7 of 11 health regions of Ontario and Saskatchewan, respectively[34]. Health regions are defined as legislated administrative areas representing geographic areas of responsibility for hospital boards or regional health authorities[35].

Secondary data sources were the provincial medical associations and health insurance boards of the provinces included in this study. These data sources provided health system level information on provincial per capita numbers of gastroenterologists and general practitioners and provincial endoscopist fees.

### Study population

A total of 130,000 respondents were surveyed from health regions of all provinces and territories between January and December 2003[18]. Study subjects were respondents who completed the CRC screening module and were at average-risk for developing CRC. For the purpose of the present study, average risk respondents were defined as those who were at least 50 years of age, without past or present CRC.

### Independent study variables

*Socio-demographic and lifestyle characteristics *included age, sex, education, country of birth, cultural/racial origin, daily servings of fruits and vegetables, number of alcoholic drinks in the past week, household income, employment status and participation in physical activities in the past 3 months. *Clinical characteristics *included self-perceived general health, smoking status, chronic conditions, having a regular physician, bowel disease (Crohn's disease, ulcerative colitis), receipt of flu shots (over lifetime). *Psychosocial characteristics *included self-perceived mental health, life satisfaction, self-perceived stress and self-perceived work stress. *Environmental characteristics *included residential area, health region of residence, province, provincial per capita numbers of gastroenterologists and general practitioners in 2003, and provincial endoscopist fees (based on 2003 colonoscopy fees).

### Outcomes

Four adherence outcomes were defined based on reported use of FOBT and endoscopy (sigmoidoscopy or colonoscopy). *Ever use *was equal to 1 if the respondent ever had either an FOBT or an endoscopy and was equal to 0 otherwise. *Adherence to FOBT screening guidelines *was defined has having an FOBT within the past 2 years. *Adherence to endoscopy screening guidelines *was defined as undergoing endoscopy in the past 10 years. *Adherence to current CRC screening guidelines *was defined as having adherence to either FOBT or endoscopy as just defined. These variables were coded as binary with 1 indicating adherence and 0 non-adherence. The criteria used to define the adherence outcomes were consistent with CRC screening recommendations in place in 2003.

### Statistical analyses

Descriptive statistics were used to characterize the study population. We used Generalized Estimating Equations (GEE) regression[36] and backward elimination (Statistical Analysis System (SAS) GENMOD procedure [37]) to identify the socio-demographic, lifestyle, clinical, psychosocial and environmental correlates of the four study outcomes. The GEE approach accounts for clustered data (possible correlations of outcomes of respondents in the same health region) and for the unbalanced structure of the data, as the number of respondents varied across provinces or health regions. Province was a four-level fixed effect (Newfoundland, Ontario, Saskatchewan, British Columbia). Provincial *per capita *numbers of gastroenterologists and general practitioners and endoscopist fees (10$ increment) were treated as continuous variables. Provincial variables did not vary within province (for example provincial endoscopist fee is the same for all people in a province) and, therefore, could not be entered together with province into the same regression model. Respondents with bowel disease were excluded from the regression analyses as use of FOBT and endoscopy may have been performed for disease surveillance. Multiple imputation was performed (SAS MI procedure version 9.1.3) to account for missing variables values (28.9% of respondents had at least 1 missing value). The final models contained only significant variables (p-values around 0.05 or less). Odds ratios (OR) and 95% confidence intervals (95%CI) were obtained by exponentiating the parameter estimates. All results presented in this paper represent *weighted values*, in keeping with the regulations of Statistics Canada. Population sample weights and bootstraps weights provided by Statistics Canada were used to produce estimates for all variables. Because the CCHS Cycle 2.1 sample design was complex, sample weights were used to account for the unequal probability of selection of individuals in the different provinces into the survey. Sample weights are based on the probability of selection into the survey and adjust for possible bias resulting from non-response[38]. Two-tailed p-values less than 0.05 were considered to be statistically significant. Ethical approval from the Research Ethics Board of the Research Institute of the McGill University Health Centre and permission from Statistics Canada were obtained prior to study inception.

## Results

A total of 17,498 eligible respondents, representing 2,529,577 Canadians, comprise the study population. Table 1 presents the socio-demographic, lifestyle, clinical, psychological and environmental characteristics of the study population. The majority of respondents were: female, aged 50–64, post-secondary school graduates, born in Canada, White, physically inactive, not employed, former smokers, without bowel disease, resided in British Columbia and lived in urban areas. Most had a chronic condition, a regular medical doctor and received a flu shot. Table 2 presents rates of non-adherence. Most (58.3%) respondents had never received either FOBT or endoscopy. Specific rates of non-adherence were 84.9% for FOBT, 79.4% for endoscopy and 69.9% for current CRC screening guidelines.

**Table 1:** Socio-demographic, lifestyle, clinical, psychological and environmental characteristics of the study population (N* = 17,498)

**Characteristic**	**Category**		**N**	**%**^a^
***Socio-demographic/lifestyle***				

Sex	male		1208664	47.8
	female		1320913	52.2
Age	50–64		1479850	58.5
	65+		1049727	41.5
Education	< high school		689570	28.1
	high school grad.		487774	19.9
	post-high school		146537	6.0
	post-high school grad.		1132259	46.1
Country of birth	Canada		1727948	69.7
	other		750832	30.3
Cultural/racial origin	White		2174040	87.7
	other		303954	12.3
Household income	low-low medium		696891	33.5
	upper medium		720335	34.6
	high		661775	31.8
Fruits & vegetables	-		mean = 4.99	sd = 2.32
Number of alcoholic drinks	-		mean = 3.04	sd = 5.78
Physical activity	active		584365	24.2
	moderate		625280	25.9
	inactive		1205071	49.9
Employment status	full-time		889514	36.1
	part-time		216564	8.8
	no job		1357573	55.1

***Clinical***				

Self-perceived general health^b^	excellent – poor		mean = 2.6	sd = 1.1
Smoking status	current		399069	15.9
	former		1305483	52.0
	never		804093	32.1
Bowel disease	yes		90678	3.6
	no		2434886	96.4
Chronic condition	yes		2129661	84.4
	no		393254	15.6
Regular physician	yes		2393987	94.7
	no		134991	5.3
Flu shot	yes		1516797	62.7
	no		902214	37.3

***Psychosocial***				

Self-perceived mental health^b^	excellent – poor		mean = 2.0	sd = 0.9
Life satisfaction ^b^	very satis. – very dissatis.		mean = 1.7	sd = 0.8
Self-perceived stress ^b^	not at all – extremely		mean = 2.5	sd = 1.1
Self-perceived work stress^b^	not at all – extremely		mean = 2.9	sd = 1.0

***Environmental***				

Health region	43 regions		-	-
Province^c^	NF & B		163555	6.5
	ON		946484	37.4
	SK		178713	7.1
	BC		1240825	49.1
Residential area	urban		2002811	79.2
	rural		526765	20.8

***Provincial***	**NF & B**	**ON**	**SK**	**BC**

Number gastroenterologists^d^	1.55	1.23	0.40	1.00
Number general practitioners^e^	1.24	0.83	1.05	1.10
Endoscopist fees ^f^	125$	57$	143$	179$

All numbers are weighted

*Number of respondents representing 2,529,577 Canadians

^a ^Percentage based on valid responses (excludes missing values)

^b ^Based on 5-item rating scale

^c ^NF & LB = Newfoundland and Labrador; ON = Ontario; SK = Saskatchewan; BC = British Columbia

^d ^Number gastroenterologists per 100,000

^e ^Number general practitioners per 1,000

^f ^Fees based on 2003 physician remuneration for colonoscopy performance

**Table 2:** Frequency of the four adherence outcomes (N* = 17,498)

**Outcome**	**Category**	**N**	**%**^a^
Ever use	yes	999841	41.7
	no	1395604	58.3
Adherence to FOBT screening guidelines	yes	356535	15.1
	no	2005605	84.9
Adherence to endoscopy screening guidelines	yes	490128	20.6
	no	1890047	79.4
Adherence to current CRC screening guidelines	yes	720899	30.1
	no	1673225	69.9

All numbers are weighted

*Number of respondents representing 2,529,577 Canadians

^a ^Percentage of valid responses (excludes missing values)

FOBT = fecal occult blood test

CRC = colorectal cancer

Table 3 presents the results of the multivariate models for the four CRC screening outcomes. The final models included sex, age, country of birth, ethnic/cultural background, household income, physical activity, employment, fruit and vegetable consumption, smoking status, chronic condition, having a regular physician, flu shot, self-perceived stress and province. *Ever use *was associated with older age, born in Canada, White race, high income level, active/moderate physical activity, not employed, increased consumption of fruits and vegetables, former smoker, having: a chronic disease, a regular physician, a flu shot, higher self-perceived stress and residing in Ontario. *Adherence to FOBT screening guidelines *was associated with male sex, high income level, active/moderate physical activity, not employed full-time, increased consumption of fruits and vegetables, and having: a chronic condition, a regular physician, and a flu shot. Residents of Newfoundland were less likely than those in British Columbia to adhere to FOBT screening guidelines. *Adherence to endoscopy screening guidelines *was associated with born in Canada, White race, not employed, former smoker, having: a chronic disease, a regular physician, a flu shot, higher self-perceived stress, and residing in Newfoundland or Ontario. *Adherence to current CRC screening guidelines *was associated older age, born in Canada, White race, high income level, active/moderate physical activity level, not employed, former smoker, having: a chronic condition, a regular physician, a flu shot, higher self-perceived stress and residing in Ontario. When provincial variables rather than province were included in the models, lower endoscopist fees were linearly associated with adherence to endoscopy screening guidelines (data not shown).

**Table 3:** Socio-demographic, lifestyle, clinical, psychological and environmental characteristics that discriminate between the four adherence outcomes (N = 16,791* **)

		**Ever use**			**Adherence to FOBT screening guidelines**			**Adherence to endoscopy screening guidelines**			**Adherence to current CRC screening guidelines**		
**Characteristic**	**Category**	**OR**	**95%CI**	**p value**	**OR**	**95%CI**	**p value**	**OR**	**95%CI**	**p value**	**OR**	**95%CI**	**p value**

Sex	female				0.71	0.57–0.88	**0.0021**						
	male				1		.						
Age	50–64	0.82	0.72–0.93	**0.0023**	0.93	0.79–1.10	0.4002	0.92	0.80–1.06	0.2648	0.89	0.82–0.98	**0.0168**
	65 +	1			1			1			1		
Country of birth	Canada	1.12	1.00–1.26	**0.0599**				1.27	1.13–1.43	**0.0001**	1.13	1.01–1.27	**0.0279**
	other	1						1			1		
Cultural/racial origin	White	1.64	1.29–2.07	**<.0001**				1.33	1.05–1.70	**0.0192**	1.42	1.12–1.81	**0.0043**
	other	1						1			1		
Household income	low	0.87	0.78–0.97	**0.0113**	0.82	0.72–0.93	**0.0016**				0.84	0.76–0.93	**0.0005**
	medium	0.99	0.90–1.09	0.8629	0.93	0.81–1.06	0.2623				0.96	0.88–1.04	0.2724
	high	1			1						1		
Physical activity	active	1.25	1.09–1.43	**0.0017**	1.37	1.15–1.62	**0.0003**				1.25	1.08–1.45	**0.0035**
	moderate	1.23	1.13–1.34	**<.0001**	1.21	1.04–1.41	**0.0143**				1.20	1.08–1.33	**0.0005**
	not active	1			1		.				1		
Employment	full-time	0.70	0.61–0.81	**<.0001**	0.73	0.62–0.87	**0.0004**	0.83	0.73–0.95	**0.0063**	0.76	0.67–0.87	**<.0001**
	part-time	0.87	0.73–1.05	0.1516	0.84	0.69–1.03	0.0910	0.87	0.69–1.11	0.2568	0.81	0.68–0.98	**0.0282**
	no job	1			1			1			1		
Fruits & vegetables	-	1.03	1.01–1.05	**0.0025**	1.05	1.03–1.06	**<.0001**				1.02	1.00–1.05	0.0667
Smoking status	current	0.91	0.78–1.06	0.2077	0.85	0.65–1.11	0.2292	0.84	0.71–1.00	0.0537	0.83	0.70–0.98	**0.0241**
	former	1.21	1.11–1.33	**<.0001**	1.10	0.94–1.29	0.2496	1.20	1.06–1.36	**0.0042**	1.19	1.07–1.33	**0.0021**
	never	1			1			1			1		
Chronic condition	yes	1.49	1.28–1.74	**<.0001**	1.32	1.11–1.57	**0.0021**	1.48	1.20–1.82	**0.0002**	1.43	1.25–1.63	**<.0001**
	no	1			1			1			1		
Regular physician	yes	1.81	1.50–2.18	**<.0001**	2.68	1.77–4.04	**<.0001**	1.91	1.46–2.50	**<.0001**	2.39	1.87–3.03	**<.0001**
	no	1			1			1			1		
Flu shot	yes	1.61	1.47–1.78	**<.0001**	1.59	1.39–1.82	**<.0001**	1.51	1.37–1.66	**<.0001**	1.55	1.41–1.70	**<.0001**
	no	1			1			1			1		
Self-perceived stress	-	1.10	1.05–1.15	**<.0001**				1.07	1.01–1.12	**0.0132**	1.06	1.01–1.11	**0.0162**
Province^a^	NF & B	0.96	0.78–1.17	0.6547	0.61	0.37–0.99	**0.0463**	1.35	1.05–1.73	**0.0189**	1.02	0.84–1.23	0.8474
	ON	1.28	1.03–1.59	**0.0284**	1.39	0.85–2.28	0.1928	1.42	1.22–1.64	**<.0001**	1.43	1.13–1.80	**0.0025**
	SK	1.14	0.76–1.73	0.5190	1.27	0.74–2.16	0.3821	1.19	0.92–1.53	0.1803	1.18	0.76–1.82	0.4602
	BC	1			1			1			1		

Significant p-values are bolded

*Number of respondent representing 2,438,766 Canadians

^a ^NF & LB = Newfoundland and Labrador; ON = Ontario; SK = Saskatchewan; BC = British Columbia

**Respondents with bowel disease were excluded from analysis

## Discussion

This population-based study showed low utilization of CRC screening in Canadians, with 58% of the study population having never received either FOBT or endoscopy. Of importance, 70% of respondents were not adherent to current CRC screening guidelines; specifically, 85% had not received FOBT within the last 2 years and 80% had not received endoscopy within the last 10 years. Not only were the majority of Canadians not screened for CRC, but also most of those who had undergone these procedures did not do so according to current recommendations. Our results mirror those reported by other Canadian studies. For example, in 2005, 77% of surveyed Ontarians 50 years of age and over without CRC had never been screened for CRC[8], and close to 80% of screen-eligible Ontario beneficiaries failed to receive CRC screening during a 6 year follow-up period[7].

Socio-demographic characteristics were associated with adherence to FOBT screening guidelines in this and other studies, including male sex[23,30,39-41] and highest income level [42], which were associated with increased adherence. Whereas other studies showed an opposite trend concerning gender differences, with women being more likely to receive FOBT and/or less likely to receive the invasive large bowel procedures [43-46], it nonetheless remains that adherence to CRC screening guidelines is low. In the present study, working full-time was associated with poorer adherence outcomes, perhaps because of the time commitments involved in preparing for and performing CRC screening[40,47]. FOBT requires that people adhere to dietary restrictions prior to testing while endoscopy involves a preparation of bowel cleaning and, often, sedation during the procedure. Alternatively, persons who were not employed may have been ill and sought more frequent health care compared to those who were employed, increasing the chance of being sent for screening. Individuals who were born in Canada and White were more likely to adhere to endoscopy screening guidelines, similar to other studies[31,46,48,49]. Collectively, these findings reinforce the need to tailor CRC screening interventions to underserved groups in order to decrease gaps in CRC screening utilization.

Healthy lifestyle behaviors as well as factors that motivate people to seek health care were associated with adherence to CRC screening guidelines. For example, having a regular physician, a chronic condition and a flu shot were associated with all adherence outcomes while increased physical activity, smoking cessation and increased consumption of fruits and vegetables were associated with at least two outcomes. Because people who visit their physicians less frequently may be at risk for not receiving recommended preventive health care, referrals or invitations for CRC screening should come from a source that is independent of physician visits, such as the government. Moreover, as public awareness has contributed to the adoption of healthy lifestyle practices, increasing public knowledge of CRC screening may be a vehicle for improving CRC screening rates.

Environmental characteristics such as lower endoscopist fees were only modestly associated with adherence to endoscopy screening guidelines, suggesting that financial incentives do not influence physician referral for endoscopy, possibly because referrals are delivered by primary care physicians. The per capita numbers of gastroenterologists and family physicians did not influence any of the four CRC screening study outcomes. Inasmuch as number of practicing physicians varied widely among provinces, our findings suggest that availability and access to screening services, particularly to gastroenterologists that perform colonoscopy, do not influence adherence to CRC screening guidelines in Canada. This finding is further underscored by the lack of association between residential area (urban/rural) and the adherence outcomes. Whereas this finding parallels that found in Alberta[9], it is in contrast to others that suggest that service availability, especially for the invasive large bowel procedures, affects utilization [46,50].

Systematic differences in CRC screening utilization were observed among the four provinces. Respondents in British Columbia were least likely to adhere to endoscopy and to current screening guidelines, while respondents in Newfoundland were least likely to have ever used or adhere to FOBT screening guidelines. These findings were independent of both financial incentives and number of practitioners who could offer or perform screening, suggesting provincial incentives or disincentives to providing CRC screening to residents may be operative. In fact, of the four provinces included in this study, only Ontario, which had the highest proportion of adherent residents, recently launched a CRC screening program [51]. Likewise Alberta and Manitoba have also recently launched CRC screening programs[52,53].

Across provinces, self-perceived stress was independently associated with ever use of FOBT and endoscopy, adherence to endocosopy screening guidelines and adherence to current CRC screening guidelines. It may well be that, like individuals with a regular physician or a chronic condition, stressed individuals seek frequent health care that increases their chance for receiving preventive health services. Similarly, patients who are among the worried well, who perceive themselves to be at high risk for CRC, may ask their physicians to be referred for screening. Whereas the reverse scenario may also be true, that individuals may be stressed as a psychological consequence of screening, this seems less plausible because self-perceived stress was not associated with adherence to FOBT screening guidelines, a test with a high false positive rate[54].

Study limitations and strengths should be considered in the interpretation of our results. The major limitation is cross-sectional design that precludes determining the directionality of associations between variables. Individuals who underwent sigmoidoscopy beyond the recommended 5-year interval were considered adherent to endoscopy as the survey did not distinguish between sigmoidoscopy and colonoscopy. Survey data were obtained by self-report and not validated. All procedural indications, including procedures done as part of a regular check-up/routine screening, or because of age, race, family history of CRC or follow-up to a previous problem, were considered in the adherence outcomes. Procedures that may have been diagnostic (i.e. follow-up to a previous problem) were not excluded because once the procedure is performed, it would not have to be repeated for another 2 (FOBT) or 10 (endsocopy) years regardless of the initial indication. Lastly, although some of the demographics of our sample are similar to those of the entire Canadian population (% female: 52 vs. 51; % rural residents: 21 vs. 19) our findings may not be generalizable to provinces that were not included in the CCHS survey or where only some health regions were included. Study strengths include the following: 1) adherence was defined broadly so as not to exclude individuals screened according to U.S. guidelines; 2) the study population was restricted to average-risk individuals, the group targeted for CRC screening; 3) use of a biopsychosocial approach to examine relationships between individual and health system level factors and adherence to CRC screening guidelines, 4) inclusion of health system level variables obtained from provincial organizations to enhance interpretability of geographical variation in utilization of CRC screening; and 5) findings are likely to be representative of current practice because only 3.2% of Canadians move out of province annually [55].

## Conclusion

In conclusion, only 30% of Canadians 50 years of age and older received FOBT and endoscopy according to current CRC screening guidelines whereas 58% had never received either procedure. Healthy lifestyle behaviors and characteristics that motivate people to seek health care were associated with adherence to CRC screening guidelines. By comparison, information on provincial level system factors, such as number of clinicians and endoscopy costs did not contribute substantially to adherence. Inasmuch as healthier people and those who did not seek health care were at risk for non-adherence to CRC screening guidelines, invitations for CRC screening should come from a source that is independent of physician visits in order to reduce CRC screening disparities in the population. These findings may assist decision and policy-makers in planning for provision of preventive services for CRC. Moreover, researchers may use the findings to tailor and evaluate interventions aimed at improving rates of CRC screening.

## Abbreviations

CCHS Canadian Community Health Survey

CRC colorectal cancer

FOBT fecal occult blood test

OR odds ratio

CI confidence interval

## Competing interests

The author(s) declare that they have no competing interests.

## Authors' contributions

MJS conceived of the study and oversaw the conduct of the study. MJS and AC designed the study. MJS and CF wrote the manuscript. AD performed the analysis under the supervision of AC and contributed to the initial draft of the manuscript. All authors read and approved the final manuscript.

## Pre-publication history

The pre-publication history for this paper can be accessed here:


